# P-1220. Humanizing Plasma and Pulmonary Epithelial Lining Fluid (ELF) Exposures of Cefiderocol in Standardized Murine Lung Infection Model

**DOI:** 10.1093/ofid/ofae631.1402

**Published:** 2025-01-29

**Authors:** Andrew J Fratoni, Alissa Padgett, Erin Duffy, David P Nicolau

**Affiliations:** Hartford Hospital, Hartford, Connecticut; Hartford Hospital, Hartford, Connecticut; CARB-X, Boston, Massachusetts; Hartford Hospital, Hartford, Connecticut

## Abstract

**Background:**

The clinical translation of preclinical *in vivo* murine infection models can be enhanced by humanizing exposures. However, humanizing plasma exposures does not ensure equivalent exposures at other target sites, such as the ELF, due to interspecies differences in penetration. We developed both plasma and ELF human simulated regimens (HSRs) of cefiderocol in a standardized murine lung infection model.

**Table 1.**
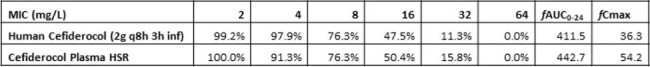
Cefiderocol %free time>MIC plasma profiles of mouse and man

**Methods:**

In accordance with the collaboration for prevention and treatment of MDR bacterial infections (COMBINE) murine lung infection model, specific pathogen-free, 6-8 week old female CD-1 mice were rendered neutropenic and received a single 5 mg/kg IP injection of uranyl nitrate 3 days prior to inoculation. Under isoflurane anesthesia, mice were inoculated with 0.05 mL of a ∼10^8^ CFU/mL bacterial suspension of *Klebsiella pneumoniae* via intranasal route. A historic cefiderocol plasma HSR from a murine thigh model was used as a baseline regimen. Two hours after inoculation, cefiderocol HSRs were administered. Groups of 6 mice were euthanized at predefined timepoints throughout the dosing interval for plasma and bronchoalveolar fluid collection. Samples were assayed by LC-MS/MS and urea correction was used to determine ELF concentrations. Murine plasma protein binding (32%) was determined previously and used to calculate free drug. Pharmacokinetic models for both plasma and ELF were fitted to the data in Phoenix WinNonlin and HSRs were mathematically altered and subsequently confirmed in the model.

**Figure 1.**
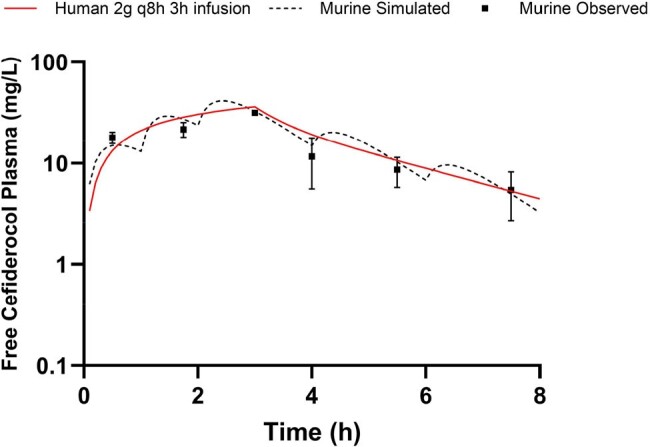
Free cefiderocol plasma concentration-time profiles in humans receiving 2g q8h as 3h infusion and mice receiving a human-simulated regimen.

**Results:**

Cefiderocol 2g q8h 3h infusion plasma exposures were recapitulated in the murine model with 5, 7.5, 10, 3.5, and 1 mg/kg at 0, 1, 2, 4, and 6h, administered every 8h, as presented in Table/Figure 1. ELF penetration for cefiderocol was greater in infected mice relative to man, necessitating a dosage reduction to 3.75, 5, 6.25, 1.75, and 1 mg/kg at 0, 1, 2, 4, and 6h, administered every 8h, to humanize ELF exposures (Figure 2).

**Figure 2.**
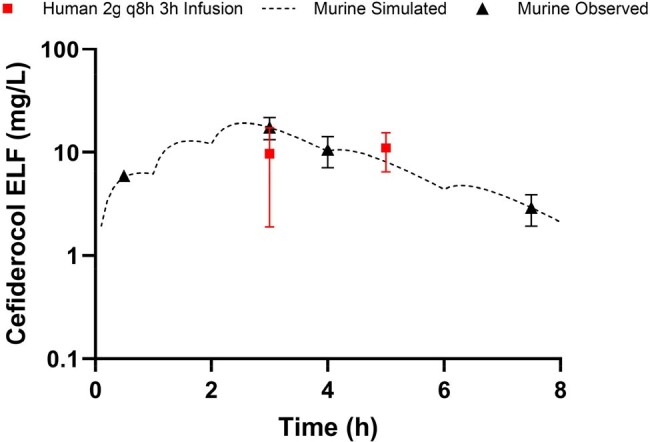
Cefiderocol pulmonary epithelial lining fluid concentration-time profiles in humans receiving 2g q8h as 3h infusion and mice receiving a human-simulated regimen.

**Conclusion:**

Plasma and ELF HSRs for cefiderocol were developed and validated in the COMBINE murine neutropenic lung infection model. Cefiderocol ELF penetration was greater in mice than man, underscoring the importance of understanding interspecies differences in target site penetration for bench-to-bedside translation.

**Disclosures:**

**Andrew J. Fratoni, PharmD**, InsightRX: Grant/Research Support **Erin Duffy, PhD**, CARB-X: Employee **David P. Nicolau, PharmD**, CARB-X: Grant/Research Support|Innoviva: Grant/Research Support|Innoviva: Honoraria|Merck: Advisor/Consultant|Merck: Grant/Research Support|Merck: Honoraria|Pfizer: Advisor/Consultant|Pfizer: Grant/Research Support|Pfizer: Honoraria|Shionogi: Advisor/Consultant|Shionogi: Grant/Research Support|Shionogi: Honoraria|Venatorx: Grant/Research Support

